# A new earwig of the genus *Echinosoma* from Penang Island, Peninsular Malaysia, with notes on the taxonomic and nomenclatural problems of the genus *Cranopygia* (Insecta, Dermaptera, Pygidicranidae)

**DOI:** 10.3897/zookeys.636.10592

**Published:** 2016-11-24

**Authors:** Yoshitaka Kamimura, Masaru Nishikawa, Chow-Yang Lee

**Affiliations:** 1 Department of Biology, Keio University, 4-1-1 Hiyoshi, Yokohama 223-8521, Japan; 2 Urban Entomology Laboratory, Vector Control Research Unit, School of Biological Sciences, Universiti Sains Malaysia, Minden 11800, Penang, Malaysia; 3 Entomological Laboratory, Faculty of Agriculture, Ehime University, Matsuyama, 790-8566, Japan

**Keywords:** Cranopygia pallidipennis, Cranopygia similis, Echinosoma roseiventre, south-east Asia

## Abstract

The pygidicranid earwigs (Dermaptera) of Penang Island, Peninsular Malaysia were re-examined based on material collected in extensive field surveys in 2012–2013 and 2015. *Echinosoma roseiventre* Kamimura & Nishikawa, **sp. n.** is described and illustrated, and *Cranopygia pallidipennis* (de Haan, 1842) is reported from the island for the first time. The taxonomic and nomenclatural problems of the genus *Cranopygia* *sensu* Hincks (1959) [A Systematic Monograph of the Dermaptera of the World. Part II. Pygidicranidae excluding Diplatyinae. British Museum (Natural History)] are also discussed. For the members of the subfamily Pygidicraninae from Indo-Austral and Oriental regions, the system, definitions of genera, and key of Hincks (1959) are followed. The genus *Mucrocranopygia* Steinmann, 1986 is synonymized with *Cranopygia* Burr, 1908. A key to the males of small *Echinosoma* from the Oriental region is provided.

## Introduction

Penang Island (Pulau Pinang) is a 299-km^2^ island located in the Straits of Malacca, approximately 5 km from the western coast of the mainland of Peninsular Malaysia. Thirty-one species of Dermaptera (earwigs) from this small tropical island are reported, based on an extensive field survey conducted in 2012–2013 (Kamimura et al. 2016), including an undescribed species of the genus *Echinosoma* Audinet-Serville, 1839 (Pygidicranidae). An additional field survey by the first author (YK) in 2014 resulted in the discovery of a species from the genus *Cranopygia* Burr, 1908 (Pygidicranidae) *sensu* Hincks (1959), which was not collected during the 2012–2013 survey (Kamimura et al. 2016). *Cranopygia similis* (Zacher, 1911) was recorded from “Penang” (Burr 1910, Hincks 1959) in the early 20^th^ century, although whether it was collected on the island or from the mainland state of Penang is unclear. Based on a comparison of the samples collected during our surveys with material preserved in museums, the identity of *Cranopygia* from Penang Island is discussed, as well as the taxonomic and nomenclatural problems of the genus *Cranopygia* *sensu* Hincks (1959).

## Methods

An extensive field survey of earwigs was conducted on Penang Island from March 2012 to March 2013 (see Kamimura et al. 2016 for details). Based on the samples collected during this survey a new species of *Echinosoma* is described. The type material of the new species and some representative samples collected during this study will be deposited in the collections of the Osaka Museum of Natural History (OMNH; Osaka, Japan) and the Lee Kong Chian Natural History Museum (LKCNHM; Singapore).

An additional field survey was conducted by YK on 9–13 March, 2015, during which time two *Cranopygia* samples were collected from Bukit Jambul (5.348821N, 100.285692E). The site is a hill with a maximum elevation of approximately 200 m a.s.l that is covered with plantations of rubber, durian, banana, and other fruit trees, and is surrounded by secondary forests. A nymphal sample collected this location was reared to adulthood in the laboratory. For comparison, we examined specimens of *Cranopygia* species from Manchester Museum (MM) and the Natural History Museum (NHM), UK, and the entomological specimen collections of the School of Biological Sciences, Univerisiti Sains Malaysia, Penang, Malaysia.

Male and female genitalia removed from the examined specimens were mounted in Euparal (Waldeck GmbH & Co. KG, Münster, Germany) between two cover slips, and attached to the pin of the respective specimen. The terminologies of Klass (2003) and Kamimura (2014) are used for female and male genital structures, respectively.

## Taxonomy

### Genus *Echinosoma* Audinet-Serville, 1839

#### Echinosoma roseiventre

Taxon classificationAnimaliaDermapteraPygidicranidae

Kamimura & Nishikawa sp. n.

http://zoobank.org/A1DA37A5-838E-4B46-A5A1-977893C9460A

Figs 1a , 2–6 , 7–9

Echinosoma sp.: Kamimura et al. 2016: 240, figs 9, 10.

##### Diagnosis.

*Echinosoma roseiventre* sp. n. is a small species less than 8 mm including the forceps. This species differs from all other similar sized species of *Echinosoma* with the combination of the following characters: abdomen uniformly reddish brown or rosy without a distinct pattern; ultimate tergite not pubescent, but with small rounded swellings; pygidium broader than long; virga very long, more than five times longer than parameres, tubular and simple.

##### Description.

**Holotype (male)**: length of body (without forceps): 7 mm. Length of forceps: 0.9 mm. Head width: 1.5 mm. Pronotum width: 1.6 mm. Pronotum length: 1.1 mm.

**Figure 1.**
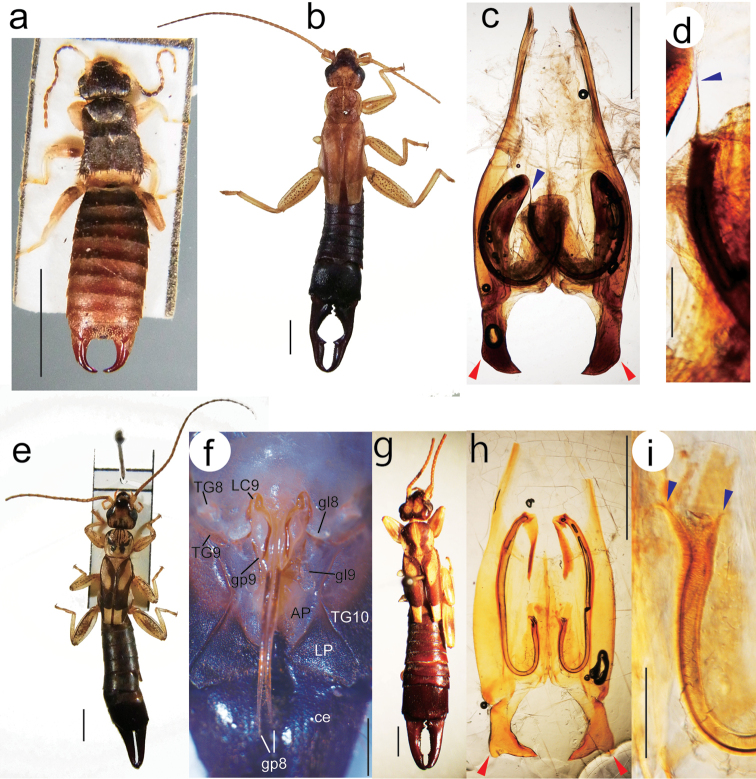
Holotype (male) of *Echinosoma roseiventre* sp. n. (**a**), a male (**b–d**) and a female (**e–f**) of *Cranopygia pallidipennis* from Penang Island, and a male of *Cranopygia similis* from Java (MM No. 3639) (**g–i**). (**a, b, e, g**) habitus; (**c, d, h, i**) male genitalia; (**f**) female genitalic region and ovipositor. The red and blue arrowheads indicate the expanded outer angle of the parameres (**c**) and the distal process of the virgae (**c, d, i**), respectively. Abbreviations: AP, anal plate; ce, cercus (=forceps); gl8, gonoplac (=coxal lobe) VIII; gl9, gonoplac (=coxal lobe) IX; gp8, gonapophysis VIII; gp9, gonapophysis IX; LC9, laterocoxa IX; LP, lateral plate; TG8–TG10, tergum VIII–X. Scale bars: 3 mm in **a, b, e**, and **g**; 1 mm in **c, f** and **h**; 200 µm in **d** and **i**.

**Figures 2–6.**
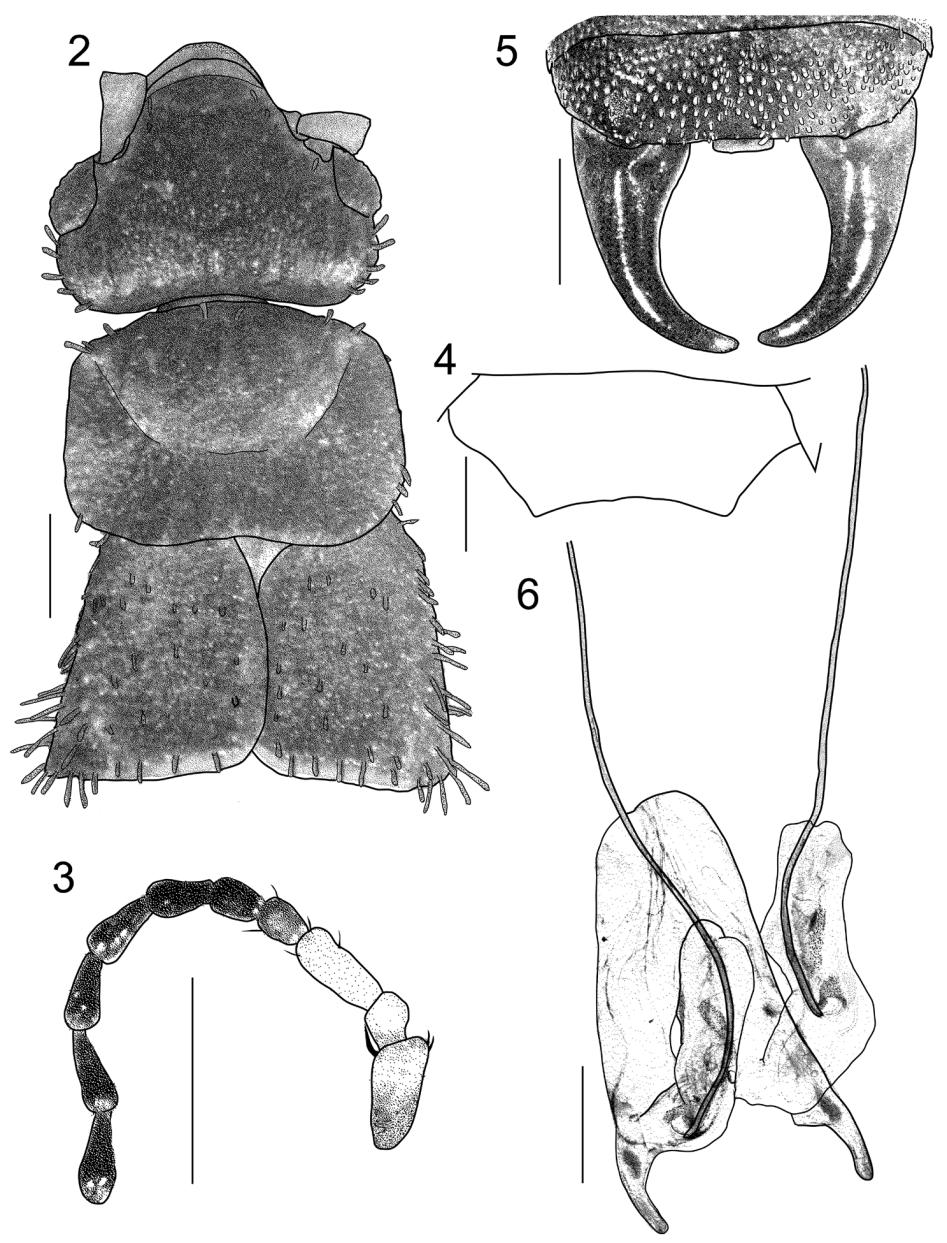
*Echinosoma roseiventre* sp. n. Holotype (male) **2** Head and thorax **3** The basal part of left antenna **4** Penultimate sternite (pubescence omitted) **5** Ultimate tergite and forceps **6** Genitalia (before mounting in Euparal). Scale bars: 0.5 mm.

**Figures 7–9.**
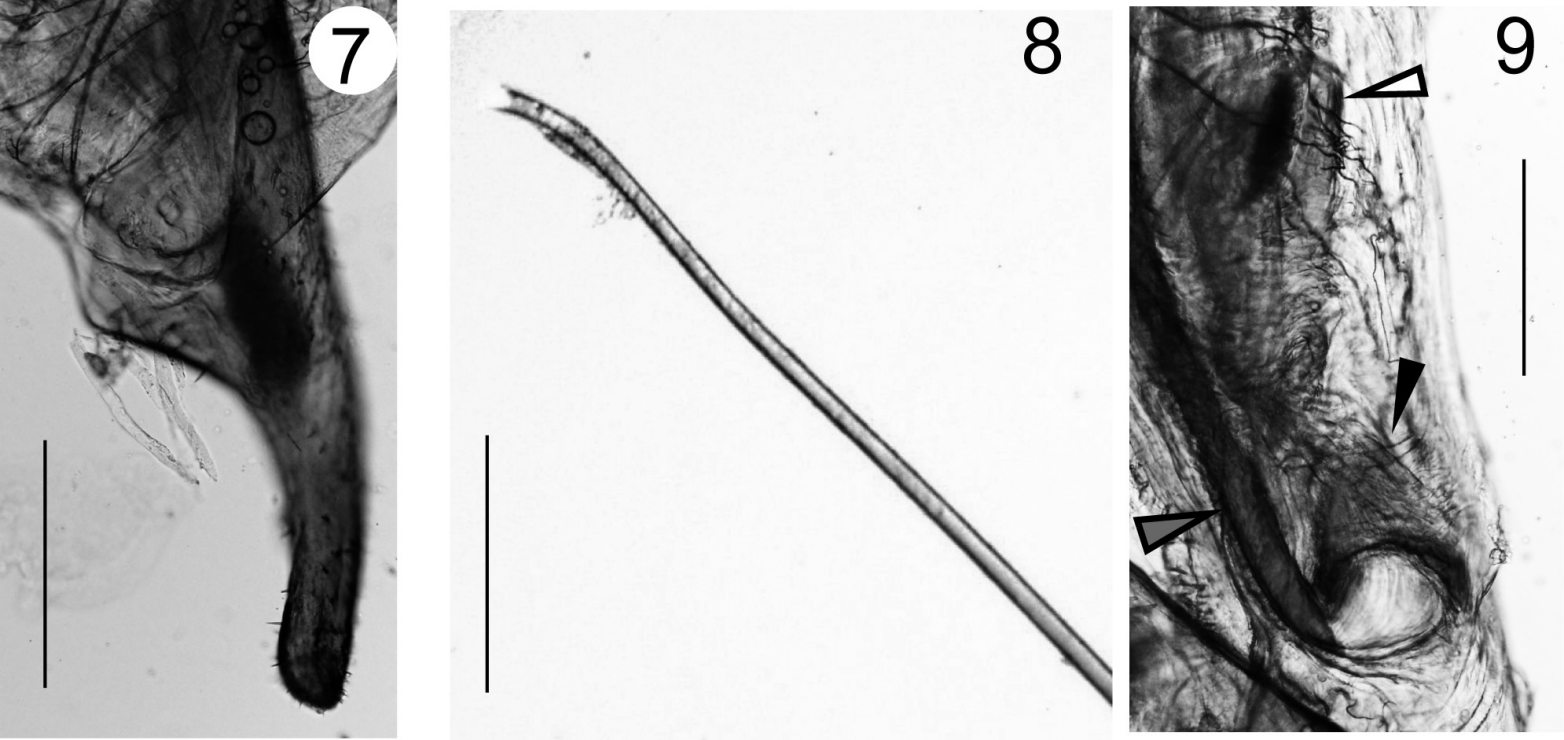
*Echinosoma roseiventre* sp. n. Holotype (male). **7** Right paramere **8** The tip of right virga **9** The base of right virga (indicated by the gray arrowhead) with the funnel-shaped sclerite (indicated by the solid arrowhead) and the long ellipse sclerite (indicated by the open arrowhead). Scale bars: 200 µm.

*Color*: General body color dull smoky black but abdomen, especially caudal part, pygidium, and forceps reddish brown or rosy (Fig. 1a). Mouth parts brownish. Antennae dark brown except for first three segments dirty white. Legs dirty white but femora with a broad fuscous band near the base. Caudal margin of tegmina with distinct, narrow whitish band. First abdominal segment whitish. *Body* covered with obtuse bristles sparsely. *Head* (Fig. 2) slightly broader than long; frons convex; transverse and median suture indistinct; caudal margin feebly emarginated in middle. Antennae (Fig. 3); 17 segments (left side partly broken, 16 segments remaining), segments mostly stout; 1^st^ expanded apically, nearly half long as the distance between antennal bases; 2^nd^ short, quadrate, almost as long as broad; 3^rd^ long, twice as long as broad; 4^th^ and 5^th^ short, as long as broad; 6^th^ and beyond gradually becoming longer and narrowing basally rendering some segments subpyriform. Eyes long, approx. as long as the post-ocular length. Post-ocular margin with a row of five long bristles. *Pronotum* (Fig. 2) broader than long; surface rough; sides rounded; frontal and caudal angles weakly and strongly rounded, respectively; caudal margin convex with distinct emargination in middle; prozona distinctively raised; median sulcus week but visible; row of long bristles on frontal and lateral margins. *Tegmina* almost as long as pronotum; surface rough; humeral angle weak and anal angle shortly rounded off to show a small, triangular scutellum; caudal margin obliquely truncate, outer and caudal margins with long bristles. *Hind wings* wanting. *Legs* stout; femora not compresed, ecarinate; arolium small; hind tarsi with 1^st^ segment longer than the third. *Abdomen* stout, more or less parallel-sided, except first three segments narrowed; sides of segments almost straight; tergites with scattered granules or very short obtuse bristles with whitish apex; first two tergites and lateral sides of 3^rd^ tergites onward with long bristles near caudal margins. Penultimate sternite (Fig. 4) transverse, narrowed posteriorly with caudal margin being nearly half of the anterior, widely emarginated. Ultimate tergite (Fig. 5) transverse, with small rounded swellings above the base of forceps; caudal margin almost straight. *Pygidium* short, rectangular, transverse. *Forceps* (Fig. 5) short, strongly curving inwards, tapering apically; surface, smooth at tips. *Genitalia* (Figs 6–9) with slender, finger-like parameres with obtuse tips and broad base (Fig. 7); penis lobe almost twice length of parameres; virga very long, more than five times longer than parameres, tubular and simple (Figs 6, 8); penis lobes also enclose a funnel-shaped sclerite at the base of virga, and a long ellipse sclerite distally (Fig. 9).

**Paratype (male).** Length of body (without forceps), 6.5 mm; length of forceps, 0.8 mm; head width, 1.2 mm; pronotum width, 1.2 mm; pronotum length, 0.8 mm. Antennae broken, five (right) and eleven (left) segments remaining. Tegmina longer, approx. 1.5 times longer than pronotum. Penultimate sternite not strongly narrows posteriorly, almost rectangular.

**Female.** Unknown.

##### Type series.

Holotype: 1 male (genitalia mounted in Euparal between two coverslips and attached to the pin of the specimen), Bukit Jambul, Penang Island, West Malaysia, 27.XI.2012, Y. Kamimura leg. [OMNH]. Paratype: 1 male (genitalia mounted in Euparal between two coverslips and attached to the pin of the specimen), same locality as holotype, 24.VI.2012 (8.VII.2012 emerged from a nymph), Y. Kamimura leg. [LKCNHM].

##### Distribution.

Penang Island, Peninsular Malaysia

##### Etymology.

The specific epithet refers to the characteristic rosy abdomen of this new species.

##### Remarks.

*Echinosoma roseiventre* sp. n. is very close to *Echinosoma andamanensis* Srivastava, 1988, described from India. Currently these two species can only be distinguished by differences in the length of the virgae (shorter than five times the parameres in *Echinosoma andamanensis*), the shape of the pygidium (longer than broad in *Echinosoma andamanensis*), and body coloration (*Echinosoma andamanensis* is generally dull smoky black but the abdomen, pygidium, and forceps are shiny; Srivastava 1988).

In addition to the species listed in the key below, *Echinosoma rufomarginatum* Borelli, 1931, which Hincks (1959), Steinmann (1986) and Srivastava (1988) treated as a doubtful species, also has a small body size (body length with forceps of ~11 mm; Hincks 1959). However, according to the original description by Borelli (1931), the male penultimate sternite of this species has a very deep emargination on the caudal margin. The male genitalia of *Echinosoma burri* Hincks, 1959, recorded from Java and Sumatra, are very similar to those of *Echinosoma roseiventre* sp. n., but the body size is much larger (male body length with forceps of 18–20 mm; Hincks 1959).

### Key to the small *Echinosoma* species (body length + forceps = 10 mm or less) from the Oriental Region (males only)

**Table:** 

1	Abdomen with distinct pattern consisting of three light longitudinal stripes or series of spots	**2**
–	Abdomen more or less uniformly colored, without distinct pattern	**4**
2	Sides of pronotum rounded. Virga almost straight	***Echinosoma affine* Hincks, 1959**
–	Sides of pronotum straight, parallel.	**3**
3	Virga slightly undulate	***Echinosoma trilineatum* Borelli, 1921**
–	Virga very long, convoluted	***Echinosoma sarawacense* Borelli, 1959**
4	Pygidium characteristic, forming a large rounded lobe, filling the space between forceps, produced into a sharp pointed spine above posteriorly	***Echinosoma maai* Srivastava, 2003**
–	Pygidium normal, without a sharp pointed spine above posteriorly	**5**
5	Ultimate tergite with long pubescence	**6**
–	Ultimate tergite setose or with very short, sparse, adpressed setae	**8**
6	Virga not longer than penis lobe	***Echinosoma sumatranum* (de Haan, 1842)**
–	Virga longer than penis lobe	**7**
7	Virga convoluted	***Echinosoma convolutum* Hincks, 1959**
–	Virga almost straight, not convoluted	***Echinosoma komodense* Bey-Bienko, 1970**
8	Virga not longer than penis lobe	**9**
–	Virga longer than penis lobe	**10**
9	Penis lobe with long strong bristles (or toothed pad) beside virga	***Echinosoma setulosum* Hincks, 1959**
–	Penis lobes without long strong bristles (or toothed pad)	***Echinosoma parvulum* Dohrn, 1863**
10	Virgae shorter than five times of parameres in length. Pygidium longer than broad	***Echinosoma andamanensis* Srivastava, 1988**
–	Virgae longer than five times of parameres in length. Pygidium broader than long	***Echinosoma roseiventre* sp. n.**

#### Genus Cranopygia

Taxon classificationAnimaliaDermapteraPygidicranidae

Burr sensu Hincks (1955)

Cranopygia pallidipennis (de Haan, 1842)

##### Material examined.

Male, preserved in the collection of the laboratory of entomology (Makmal Entomologi), School of Biological Sciences, Universiti Sains Malaysia: Taman Rimba (Teluk Bahang Recreational Park), Penang Island, 9 XII 2009, Tan Chia Chi leg. The specimen has now been transferred to the entomological specimen collections of the School of Biological Sciences, Universiti Sains Malaysia. Two females (one emerged from nymph on 30 III 2015): Bukit Jambul (secondary forest of a rubber plantation), Penang Island, 11 III 2015, Y. Kamimura leg.

##### Comparative material examined.

*Cranopygia similis* (Zacher, 1911): Male, preserved in the collection of the Manchester Museum, the University of Manchester, England: “H. LUCHT, K. O. Blawan, 900/1500 Mr., Idjan Plateau [with unreadable handwritten characters: ? 205.39] / 3639 / Cranopygia similis (Zacher) ♂, det W. D. Hinks” [MM No. 3639].

##### Known distribution.

Malaysia (Kuala Lumpur, Bukit Kuru), Myanmar, Indonesia (Java, Sumatra, Borneo).

##### Remarks.

First record for Penang Island.

## Discussion

### Problems in the taxonomic treatment of *Cranopygia* Burr *sensu* Hincks (1955)

Within the family Pygidicranidae, the subfamily Pygidicraninae Verhoeff, 1902 is characterized by a medium to large body size (rarely less than 20 mm), antennae with 25 segments or more in which the 4^th^ and 5^th^ are wider than they are long, depressed femora, and equally developed right and left penis lobes (Burr 1915a, Hincks 1955, Steinmann 1986, Srivastava 1988). Indo–Austral and Oriental species of this subfamily are usually classified in the genus *Tagalina* Dohrn, 1863, in which the second tarsal segments are characteristically enlarged, or the genus *Cranopygia* Burr, 1908 *sensu* Hincks (1955). The taxonomy of the latter is rather unstable and unsettled. Including this group, for several species that were formerly in the genus *Pygidicrana* Audinet-Serville, 1831, Burr (1908) erected the following four genera based on differences in the shapes of the penultimate sternite, pronotum, and elytra: *Cranopygia* (type species, *Pygidicrana cumingi* Dohrn, 1863), *Pyge* (type species, *Pygidicrana modesta* de Bormans, 1894), *Dicrana* (type species, *Pygidicrana frontalis* Kirby, 1903), and *Picrania* (type species, *Pygidicrana liturata* Stål, 1855). Subsequently, Zacher (1911) established the genus *Kalocrania* (type species: *Pygidicrana marmoricrura* Audinet-Serville, 1839), to which two additional species of Oriental *Pygidicrana* were transferred, with the description of a new species. However, the species of *Cranopygia* *sensu* Burr (1908) were apparently unknown to Zacher, which resulted in a lack of agreement as to how to distinguish between *Cranopygia* and *Kalocrania* (see Hincks 1955 for more details). To settle this problem, Burr (1915a) consistently examined the male genitalia of this group for the first time, and redefined the genus *Cranopygia* based on the shape of the virga. Simultaneously, *Pyge* was synonymized with *Kalocrania*, and a new genus *Acrania* was established (type species, *Pygidicrana picta* Guérin-Méneville, 1838). Hincks (1955), who examined the genital armatures for many more species in this group, concluded that *Cranopygia*, *Kalocrania*, and *Acrania* could not consistently be distinguished based on their genital morphologies, and he later synonymized the latter two genera with *Cranopygia*, with the formation of five species groups (Hincks 1959). Several species formerly in the genus *Dicrana* were also included in *Cranopygia* by Hincks (1959).

Nearly 25 years later, Steinmann (1986) erected three new genera, *Epicranopygia* (type species: *Pygidicrana picta* Guérin-Méneville, 1838), *Mucrocranopygia* (type species: *Pygidicrana horsfieldi* Kirby, 1891), and *Paracranopygia* (type species: *Forficula pallidipennis* de Haan, 1842), for the species of *Cranopygia* *sensu* Hincks (1959) with virgae that were not straight. Srivastava (1993a) considered that the traits for diagnosing these genera (i.e., the shapes of the penis lobes and the virgae) were unstable and therefore unsuitable for generic classification. Instead, he focused on the shape of the parameres, which are robust and resistant to the artifacts of mounting, and reinstated *Acrania* for species with parameres that are neither knobbed nor hooked externally or internally (but occasionally with a slight convexity of the external apical angle).

Engel and Haas (2007), who omitted to cite Srivastava (1993a), noted that the generic names *Acrania* and *Pyge*, which Steinmann (1986) considered invalid, were available for the group containing the respective type species. Accordingly, they reinstated *Acrania* and *Pyge*, making *Epicranopygia* and *Paracranopygia* junior objective synonyms. Although they did not provide the species lists for *Cranopygia* and *Mucrocranopygia* (*sensu* Steinmann 1986), Engel and Haas (2007) followed Steinmann’s (1986) taxonomic system for the subfamily, except for the abovementioned changes in generic names.

Srivastava’s (1993a) taxonomic treatment is also problematic. He reinstated *Acrania*, the type species of which is *Pygidicrana picta* Guérin-Méneville, 1838. However, he simultaneously synonymized *Epicranopygia*, which was created with the same type species (*Pygidicrana picta*), with *Cranopygia*. According to his list of new combinations, Srivastava (1993a) transferred three species of *Epicranopygia* to *Cranopygia*, but transferred three others, including *Echinosoma picta*, to *Acrania*. Thus, the declaration of synonyms in Srivastava (1993a), and those cited in subsequent papers (Srivastava 1993b, 1995) are incorrect: Srivastava (1993a) synomyzed *Epicranopygia* **(*pars*)** and *Paracranopygia* **(*pars*)** with *Acrania* and *Cranopygia*.

Subsequently, Sakai (1996, 2000) generally followed Srivastava’s (1993a) system (and possibly the identification key), but concurrently adopted Hincks’s (1959) species-group level classification. However, instead of using the *Cranopygia siamensis* species group (Hincks 1959), he treated *Paracranopygia* as a valid subgenus for most species of *Paracranopygia* *sensu* Steinmann (1986), as well as including *Cranopygia tianshanskyi* and *Cranopygia chirurga*, which were originally described by Gorochov and Anisyutkin (1993) under the genus *Paracranopygia*.

In addition to these nomenclatural problems, recent studies have shown that the morphology of earwig virgae, particularly the length, evolves rapidly due to sperm competition, resulting in considerable variation even among very closely related congeners (Kamimura 2000, 2014, Lieshout and Elgar 2011). Therefore, although useful for species diagnosis, generic classification systems based primarily on virgal characteristics (e.g., length, convolution) likely do not reflect accurately the phylogenetic relationships. In contrast, the functional significance of male genital parameres is largely unknown for earwigs (Kamimura 2014). Nevertheless, the presence or absence of a tooth or process of the parameres, which Srivastava (1993a) proposed to distinguish *Cranopygia* and *Acrania*, is also likely an unreliable trait for the generic classification of this group. For example, male *Cranopygia vittipennis* Hincks, 1955 have a tiny process at the outer angle of the paramere, whereas a similar but weaker process is found in *Acrania luzonica* (Brindle, 1955) in the equivalent position (compare figs. 2 and 12 of Srivastava 1993a). A similar observation was made for *Cranopygia pallidipennis* from Penang Island, which is described below. Therefore, for the taxonomy of pygidicranine earwigs, we propose to follow the system, definitions of the genera, and key of Hincks (1959); that is, all of the species from Indo–Austral and Oriental regions (except for some species of *Dacnodes*) are classified either in the genera *Tagalina* (species with an enlarged second tarsal segment) or *Cranopygia* (species with a simple second tarsal segment). Accordingly, we propose to place all of the following species in the genus *Cranopygia*.

### Genus *Cranopygia* Burr and its synonyms

*Cranopygia* Burr, 1908: 384, 389 [type-species: *Pygidicrana cumingi* Dohrn, 1868 (original designation)]; 1910: 53, 61; 1911: 16, 19; 1915a: 432, 435 (*Pyge* Burr, proposed synonymy with *Cranopygia* Burr). – Townes 1945: 346 (catalogue). – Hincks 1955: 809 (*Kalocrania* Zacher and *Acrania* Burr, proposed synonymy with *Cranopygia*); 1959: 52 (revision). – Popham 1965: 132 (in key). – Brindle 1970: 647. – Sakai 1971: 12 (catalogue); 1982: 15 (list of species); 1996: 3 (list of species); 2000: 89 (in key). – Steinmann 1973a: 148 (list); 1973b: 396 (in key); 1975: 202 (in key); 1983: 56 (synopsis); 1986: 240 (revision); 1989: 122 (catalogue). – Srivastava 1988: 37 (classification same as Hincks 1959); 1993a (1992): 43 (*Epicranopygia* Steinmann and *Paracranopygia* Steinmann, proposed synonymy with *Cranopygia*); 1995: 293 (*Epicranopygia* Steinmann and *Paracranopygia* Steinmann, as synonyms of *Cranopygia*).

*Pygidicrana* (*pars*) Audinet-Serville, 1831: 30 [type-species: *Pygidicrana v-nigrum* Audinet-Serville, 1831 (Monobasic)]; 1839: 19. – Dohrn 1863: 46. – Scudder 1876: 298. – de Bormans and Kraus 1900: 15. – Kirby 1904: 4. – Burr 1908: 384; 1910: 53.

*Pyge* (*pars*) Burr, 1908: 384, 390 [type-species: *Pygidicrana modesta* de Bormans, 1894 (original designation)]; 1910: 53, 65; 1911: 16, 20; 1915a: 435. – Shiraki 1928: 3. – Townes 1945: 354 (catalogue). – Engel and Haas 2007: 19 (*Paracranopygia* Steinmann, proposed synonymy with *Pyge*).

*Dicrana* (*pars*) Burr, 1908: 384, 387 [type-species: *Pygidicrana frontalis* Kirby, 1903 (original designation)]; 1910: 53, 60; 1911: 16, 19. – Townes 1945: 347 (catalogue).

*Picrania* (*pars*) Burr, 1908: 390 [type-species: *Pygidicrana liturata* Stål, 1855 (original designation)]; 1910: 53, 63; 1911: 16, 19. – Townes 1945: 353 (catalogue).

*Kalocrania* Zacher, 1910: 105 [type-species: *Pygidicrana marmoricrura* Audinet-Serville, 1839 (original designation)]. – Zacher 1911: 335, 336. – Burr 1911: 16, 18 (*pars*), pl. 8, fig. 18 (opisthomeres); 1915a: 432, 435; 1915b: 258, fig. 1 (opisthmeres), fig. 19 (gonapophyses). – Townes 1945: 350 (catalogue).

*Acrania* Burr, 1915a: 432, 436 [Type species: *Pygidicrana picta* Guérin-Méneville, 1838 (original designation)]. – Townes 1945: 343 (catalogue). – Srivastava 1993a (1992): 44 (*Mucrocranopygia* Steinmann, proposed synonymy with *Acrania*); 1993b: 373 (*Mucrocranopygia* Steinmann and *Epicranopygia* Steinmann (*pars*), as synonyms of *Acrania*); 1995: 293 (*Mucrocranopygia* Steinmann, as synonym of *Acrania*). – Sakai 1996: 2 (list of species); 2000: 100 (in key). – Engel and Haas 2007: 19 (*Epicranopygia* Steinmann, proposed synonymy with *Acrania*).

*Epicranopygia* Steinmann, 1986: 269 (proposed new name for *Acrania* Burr, 1915) [type-species: *Pygidicrana picta* Guérin-Méneville, 1838 (original designation)]; 1989: 146 (catalogue). – Sakai 1982: 16 (list of species).

*Paracranopygia* Steinmann, 1986: 277 [type-species: *Forficula pallidipennis* de Haan, 1842 (original designation)]; 1989: 150 (catalogue). – Sakai 1982: 15 (list of species).

Cranopygia (Paracranopygia) Sakai, 1996: 4 [= siamensis-group, Hincks (1959)] (list of species); 2000: 104 (in key).

*Mucrocranopygia* Steinmann, 1986: 266 [type-species: *Pygidicrana horsfieldi* Kirby, 1891 (original designation)]; 1989: 149 (catalogue). – Sakai 1982: 15 (list of species). **New synonym**.

### List of species to be included in the genus *Cranopygia*

*Cranopygia angustata* (Dohrn, 1862); *Cranopygia appendiculata* Hincks, 1955; *Cranopygia assamensis* Hincks, 1955; *Cranopygia bakeri* (Borelli, 1921); *Cranopygia beybienkoi* Gorochov & Anisyutkin, 1993; *Cranopygia bhallaie* Kapoor, 1966; *Cranopygia bifurcata* Srivastava, 1980; *Cranopygia brindlei* Srivastava, 1988; *Cranopygia burmensis* Hincks, 1955; *Cranopygia burri* Hincks, 1955; *Cranopygia carinata* Hincks, 1959; *Cranopygia celebensis* (de Bormans, 1903); *Cranopygia chirurga* (Gorochov & Anisyutkin, 1993); *Cranopygia comata* Hincks, 1955; *Cranopygia constricta* Hincks, 1955; *Cranopygia corymbifera* Anisyutkin, 1997; *Cranopygia crockeri* Anisyutkin, 2014; *Cranopygia cumingi* (Dohrn, 1862); *Cranopygia curtula* Hincks, 1955; *Cranopygia daemeli* (Dohrn, 1869); *Cranopygia dravidia* (Burr, 1914); *Cranopygia eximia* (Dohrn, 1862); *Cranopygia fletcheri* Bharadwaj & Kapoor, 1967; *Cranopygia formosa* Hincks, 1955; *Cranopygia gialaiensis* Gorochov & Anisyutkin, 1993; *Cranopygia guttata* (Kirby, 1903); *Cranopygia horsfieldi* (Kirby, 1891); *Cranopygia imperatrix* (Burr, 1899); *Cranopygia jacobsoni* (Boeseman, 1954); *Cranopygia javana* Hincks, 1955; *Cranopygia kallipygos* (Dohrn, 1862); *Cranopygia lueddemanni* Srivastava, 1984; *Cranopygia luzonica* Brindle, 1967; *Cranopygia maculipes* Hincks, 1955; *Cranopygia manipurensis* Srivastava, 1975; *Cranopygia marmoricrura* (Audinet-Serville, 1839); *Cranopygia modesta* (de Bormans, 1894); *Cranopygia nietneri* (Dohrn, 1862); *Cranopygia nova* Anisyutkin, 2015; *Cranopygia okunii* (Shiraki, 1928); *Cranopygia ophthalmica* (Dohrn, 1862); *Cranopygia pallidipennis* (de Haan, 1842); *Cranopygia parva* Brindle, 1975; *Cranopygia philippinica* Burr, 1914; *Cranopygia picta* (Guerin-Méneville, 1838); *Cranopygia pluto* Hebard, 1923; *Cranopygia proxima* Hincks, 1959; *Cranopygia raja* (Burr, 1911); *Cranopygia rostrata* Brindle, 1970; *Cranopygia sarawacensis* Hincks, 1959; *Cranopygia sauteri* (Burr, 1912); *Cranopygia semenoffi* (Burr, 1912); *Cranopygia siamensis* (Dohrn, 1862); *Cranopygia similis* (Zacher, 1911); *Cranopygia spenceri* Srivastava, 2003; *Cranopygia steineri* Srivastava, 1993; *Cranopygia steinmanni* Srivastava, 1988; *Cranopygia tianshanskyi* (Gorochov & Anisyutkin, 1993); *Cranopygia tonkinensis* Hincks, 1955; *Cranopygia tumida* Borelli, 1931; *Cranopygia valida* (Dohrn, 1867); *Cranopygia vanderdoesi* Boeseman, 1954; *Cranopygia variegata* Brindle, 1965; *Cranopygia vicina* Hincks, 1959; *Cranopygia vietnamensis* Gorochov & Anisyutkin, 1993; *Cranopygia vitticollis* (Stål, 1855); *Cranopygia vittipennis* Hincks, 1955.

### Identification of specimens of *Cranopygia* from Penang

The external morphology, coloration, and genitalia of the male specimen collected at Taman Rimba (Teluk Bahang Recreational Park), Penang Island are very similar to those of *Cranopygia pallidipennis* (de Haan, 1842) described by de Haan (1842), Burr (1910), Zacher (1911), and Hincks (1959) (Fig. 1b-d). The external morphologies and coloration of the female specimens from Bukit Jambul, Penang Island also match the descriptions of *Cranopygia pallidipennis* (de Haan 1842, de Bormans and Kraus 1900, Zacher 1911, Hincks 1959). The female genital region was also examined for a female specimen that emerged in the laboratory (Fig. 1f). Although the female genitalia are rarely described for the genus (but see Zacher 1911; Anisyutkin 2014) and thus diagnostic features have not been established, the observed morphology (Fig. 1f) matches that described by Zacher (1911) for *Cranopygia pallidipennis*.

A male specimen of *Cranopygia* was recorded from “Penang” in the early 20th century (Burr 1910; Hincks 1959). Burr (1910) identified it as *Cranopygia siamensis* (Dohrn, 1863). Later, Hincks (1959) tentatively identified the specimen as *Cranopygia similis* (Zacher, 1911) based on features of the genitalia. However, according to Hincks (1959), the large body size (36 mm) of the specimen and the following external morphology are not typical of *Cranopygia similis*; “In the Penang male the pronotum is as broad as long, and the sides are strongly rounded; the occiput is marmorated with fuscous dots and streaks; the pronotum has the dark bands much more broken; the femora are dotted with fuscous and not longitudinally streaked; the forceps are rather longer and more curved, enclosing an oblong–ovate space.” Some of these characteristics suggest a very close affinity of the specimen to *Cranopygia pallidipennis*, but the shape of the forceps is different (Burr 1910).

*Cranopygia pallidipennis* seems to be very close to *Cranopygia similis* and can be distinguished from the latter by a larger body size; the pattern of fuscous markings on the head, pronotum, and femora (Fig. 1b, e vs. Fig. 1g); a larger space enclosed by the distal part of the forceps (Fig. 1b vs. Fig. 1g); a less pronounced convexity at the outer angle of the parameres (Fig. 1c vs. Fig. 1h); and the presence of a single, long filamentous projection at the tip of the virgae (Fig. 1d vs. Fig. 1i). The last characteristic is likely a diagnostic feature distinguishing *Cranopygia pallidipennis* from *Cranopygia similis*. Unfortunately, we could not reexamine the male specimen from “Penang” described by Burr (1910) as it is currently missing; it was not found in the collections of the NHM (including Burr’s collection) or the MM. In conclusion, our study shows that *Cranopygia pallidipennis* is a member of the contemporary earwig fauna of the island, whereas the identity of Burr’s specimen of *Cranopygia* from “Penang” requires further investigation including determining the exact location from which it was collected.

## Supplementary Material

XML Treatment for Echinosoma roseiventre

XML Treatment for Cranopygia
